# A snapshot of cancer in Chile: analytical frameworks for developing a cancer policy

**DOI:** 10.1186/0717-6287-48-10

**Published:** 2015-01-26

**Authors:** Jorge Jimenez de la Jara, Gabriel Bastias, Catterina Ferreccio, Cristian Moscoso, Sofia Sagues, Camilo Cid, Eduardo Bronstein, Cristian Herrera, Bruno Nervi, Alejandro Corvalan, Ethel V Velasquez, Pamela Gonzalez, Enrique Castellon, Eva Bustamante, Sergio Oñate, Eileen McNerney, Richard Sullivan, Gareth I Owen

**Affiliations:** Department of Public Health, Pontificia Universidad Católica de Chile, Santiago, Chile; Department of Haematology and Oncology Faculty of Medicine, Pontificia Universidad Católica de Chile, Santiago, Chile; Department of Physiology, Faculty of Biological Sciences, Pontificia Universidad Católica de Chile, Santiago, Chile; Faculty of Medicine, Universidad de Chile, Santiago, Chile; Fundación Arturo López Pérez, Santiago, Chile; Faculty of Medicine, Universidad de Concepción, Concepción, Chile; King’s College London and Institute of Cancer Policy, Kings Health Partners Cancer Centre, London, UK; Biomedical Research Consortium Chile, Santiago, Chile; Centro de Evaluación de Intervenciones en Salud (CEISALUD-UC), Santiago, Chile; Fondap ACCDiS, Pontificia Universidad Católica de Chile, Santiago, Chile; Grupo Oncológico Cooperativo Chileno de Investigación (GOCCHI), Región Metropolitana, Chile; Center UC Investigation in Oncology (CITO), Pontificia Universidad Católica de Chile, Santiago, Chile; Comisión Chilena de Energía Nuclear, Santiago, Chile; Foro Nacional de Cancer, Santiago, Chile

**Keywords:** Chile, Cancer policy, Investigation, Research and development, Statistics, Gallbladder cancer, Stomach cancer, Developing country

## Abstract

**Introduction:**

The South American country Chile now boasts a life expectancy of over 80 years. As a consequence, Chile now faces the increasing social and economic burden of cancer and must implement political policy to deliver equitable cancer care. Hindering the development of a national cancer policy is the lack of comprehensive analysis of cancer infrastructure and economic impact.

**Objectives:**

Evaluate existing cancer policy, the extent of national investigation and the socio-economic impact of cancer to deliver guidelines for the framing of an equitable national cancer policy.

**Methods:**

Burden, research and care-policy systems were assessed by triangulating objective system metrics – epidemiological, economic, etc. – with political and policy analysis. Analysis of the literature and governmental databases was performed. The oncology community was interviewed and surveyed.

**Results:**

Chile utilizes 1% of its gross domestic product on cancer care and treatment. We estimate that the economic impact as measured in Disability Adjusted Life Years to be US$ 3.5 billion. Persistent inequalities still occur in cancer distribution and treatment. A high quality cancer research community is expanding, however, insufficient funding is directed towards disproportionally prevalent stomach, lung and gallbladder cancers.

**Conclusions:**

Chile has a rapidly ageing population wherein 40% smoke, 67% are overweight and 18% abuse alcohol, and thus the corresponding burden of cancer will have a negative impact on an affordable health care system. We conclude that the Chilean government must develop a national cancer strategy, which the authors outline herein and believe is essential to permit equitable cancer care for the country.

## Introduction

### Chile: a complex demographic transition

**Figure 1:**
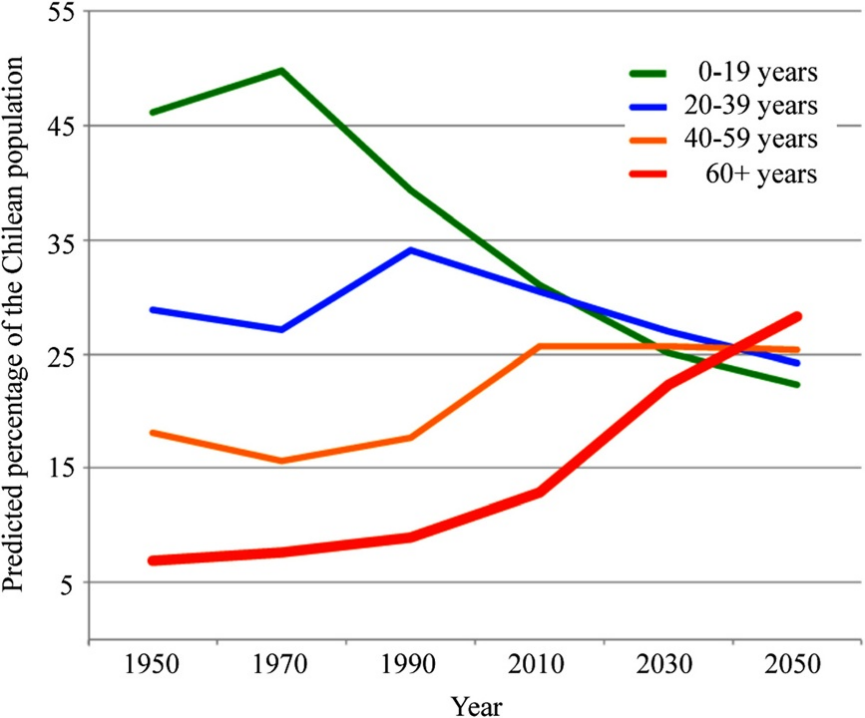
**Chile’s ageing population.** Comparison of age cohorts (%) from 1950 projected to 2050 (Data source: *CHILE: Proyecciones y Estimaciones de Población. Total País 1950–2050*[9]).

The burden of cancer worldwide is rapidly increasing, principally due the growth and ageing of the world population, coupled with an increasing adoption of cancer-causing lifestyles (eg. tobacco) in economically developing countries [1]. It is anticipated that the developing world will bear the brunt of cancer incidence and mortality in the coming years [1]. By 2030 in Latin America/Caribbean region, it is estimated that 1.7 million cases of cancer will be diagnosed, resulting in more than one million annual deaths [2]. Chile, a country of over 17.5 million people (59th most populous country on Earth) [3], of which less than 3% are immigrants [4], leads South America with the lowest percentage of it population below the poverty line. Furthermore, Chile also boasts the lowest regional international debt and is the second leading country after Brazil in international exports [5, 6]. These socioeconomic results associate with the incorporation of a westernized lifestyle and an increase in life expectancy, which have translated into an increase in cancer burden. Life expectancy in Chile currently stands at 80 years (77 years for men and 83 years for women), placing it above the average of both upper middle-income countries (74 years) and high income countries (79 years) as estimated by the World Health Organization and the Organization for Economic Co-operation and Development (OECD) [7, 8]. Half a century ago only a small percentage of the Chilean population was of pensionable age, but based on demographic statistics from the 2002 Chilean Census, this figure was estimated by the Ministry of Health to be 12.9% in 2010, rising to 22.3% in 2030 and over 28% in 2050 (Figure 1) [9]. However, recent preliminary data from the Ministry of Social Development indicates that 15.6% of Chileans were >60 years old in 2011, suggesting that Chile may be underestimating the population age structure and thus the burden of aging-related public health in the coming years [10].

Chile’s health care system is mainly public and covers some 75% of the population. A further 14% are insured by private schemes, and the remaining population covered by other mixed insurance systems. The change to a more occidental lifestyle can also be clearly observed in Chile’s socioeconomic structure. In a society stratified from A to E, where ABC are high level earners and make up 5% of the country, E is the poverty line (15% population) and D is the majority of Chileans (75%), a clear separation in cancer incidence has been reported [6].

Chile has belatedly implemented restrictions on tobacco availability to minors. According to the 2013 Health statistics, an average of 21% of each population within OECD countries use tobacco-related products. However, the recently compiled “Tobacco Atlas” by the American Cancer Society and the World Lung Foundation estimates that 37.5% of men and 33.3% of women in Chile smoke. This equates to a US$1.1 billion drain on the health service and accounts for 11% and 8% of male and female mortality, respectively. Alarmingly, 51.7% of Chile’s youth are exposed to second-hand smoke within the home, and 28% of boys under 15 and an incredible 39.9% of girls under 15 are currently smoking. This latter statistic for girls unfortunately places Chile among the world leaders in this category.

The estimated annual incidence of cancer in Chile is approximately 35,000 new cancer cases per year, with adjusted incidence rates of 226 and 180 per 100,000 male and female inhabitants, respectively [2, 11]. Cancer registries in Chile currently do not have national coverage, and thus incidence rates are principally estimated from death certificates. Approximately 22,000 annual cancer deaths are recorded, which accounts for 24% of national mortality (92,000) [9]. Mortality statistics in Chile have highlighted a cancer distribution that is both unique and of concern [11]. In 1990 the mortality rate from cancer was 107 per 100,000 Chileans. In 2007 this figure increased to 129.5 per 100,000 and then again to 137.5 per 100,000 in 2011, with rates for males and females manifesting similar upward trends. The relative burden of cancer mortality has been increasing steadily over the last decades: 8.4% in 1960, 12.1% in 1970, 15.8% in 1980, 18.1% in 1990 and 24% in 2011 [12].

Although the per capita income has more than quadrupled from US$5,000 in 1990 to over US$20,000 in 2012 (USD in Purchasing Power Parity) [13] and poverty indexes have diminished from 40% to 15% during the same period, the cancer burden profile has not transitioned to which one would expect in a pure high income country [13]. Thus, Chile fits the profile of a developing country that the UN General assembly and the World Health Organization have singled out as being the carriers of the global cancer burden in the years to come [1, 14].

Herein, we aim to quantitate the economic impact of cancer in Chile and evaluate existing cancer policy. The distribution and extent of national investigation in oncology and the socio-economic role of cancer will be assessed. An overall objective is to deliver information on the state of cancer in Chile and offer guidelines for the framing of an equitable national cancer policy.

## Methods

Epidemiological data was obtained from the literature and on-going investigation from the authors. All database references are included in detail in the reference section. Total population statistics on Chile were obtained from the World Population review, the CIA World Factbook [6] and the Chilean National Institute of Statistics (*Instituto Nacional de Estadísticas de Chile,* INE) [15] websites. Population projection data between 1950 and 2050 were based on statistics from the INE: Demographics and Vitals [9]. Further data was retrieved from the Ministry of Social Development website [16], Government of Chile: National Socioeconomic Characterization Survey, Casen 2011 [10]. World health and life expectancy data was obtained from the OECD Health Statistics database (OECD Health Data 2013) [8].

### Quantifiable data

#### Analysis of population and economic burden

Raw data for the chronic disease burden in Chile was collected from the publicly available databases from the Ministry of Health website [17]: *Proyecciones y Estimaciones de Población. Total País 1950–2050*[8] and *Informe Final Estudio de carga de enfermedad y carga atribuible, 2007*[18]. National statistics on mortality and population were obtained from the National Institute for Statistics [15]. Economic burden was based on Ministry of Health data on hospital discharges correlated to pricelist reimbursement from both the public and private (ISAPRE) sectors. The calculation of cancer in economic terms was determined by multiplying the Disability Adjusted Life Years (DALYs) due to cancer by the equivalent of the Per Capita income of the country, estimated at US$20,000 [19]. The calculation of the number of cancer related patents solicited from Chile was compiled using the keywords “Chile” together with either “tumor”, “neoplasms” or “cancer” at the National Institute of Intellectual property (Instituto Nacional de Propiedad Industrial, INAPI) [20] and World Intellectual Property Organization (WIPO) [21] webpages. The results were screened individually to validate their relevance and authenticity.

#### Clinical trials data

A registry of clinical trials was obtained from National Institute of Public Health Chile [22], the U.S. National Institutes of Health clinical trials register [23], the European Union clinical trials register [24] and through personal communication with complying hospitals and clinics.

#### Cancer research expenditures

Information on project and cancer research expenditure was obtained from the public databases from the National Commission for Science and Technology (CONICYT) [25] at the Ministry of Education and the Chilean economic development agency (CORFO) [26] within the Ministry of Economics. Additional data was obtained through the kind donation of specific funding information pertaining to biomedical consortia, science parks and personal communication with individual institutions and scientists.

#### Trends in Chilean scientific cancer-related publications and investigation

Scientific cancer-related publications (written in English and Spanish), were calculated form searches in NCBI PubMed database of the keywords “Chile” and “cancer or neoplasms” from 1970 until 2013. The relevance of the results returned from the Pubmed database was scrutinized for integrity to the search criteria. The Chilean national medical journal, the *Revista Medica de Chile*, is indexed on the NCBI Pubmed database. The increase in cancer-related presentations at national conferences (principally the *The Annual Meeting of the Chilean Society for Cellular Biology 2009–2013*) was assessed by personal participation of the authors at the meeting and a quantification of cancer related studies listed among the abstracts and oral presentations.

#### Questionnaires

Questionnaires were generated using the web-based program survey monkey and were sent to all members investigators and medics identified by the national databases of the ministries of health, economics and education. The questions were restricted to the highest degree obtained, the speciality, the publication record, financial support won and affiliated institution. Medical doctors were asked to divulge participation in international clinical trials and all investigators (MD, PhD, BSc) who had obtained national or international funding in the area of cancer were surveyed on their specific area of research. When information could not be obtained from questionnaires, academic information and area of research for individual investigators was obtained from the public databases from CONICYT and CORFO. This information was quantified and represented graphically within this publication.

## Results

### Quantifying the economic burden of cancer in Chile

**Figure 2:**
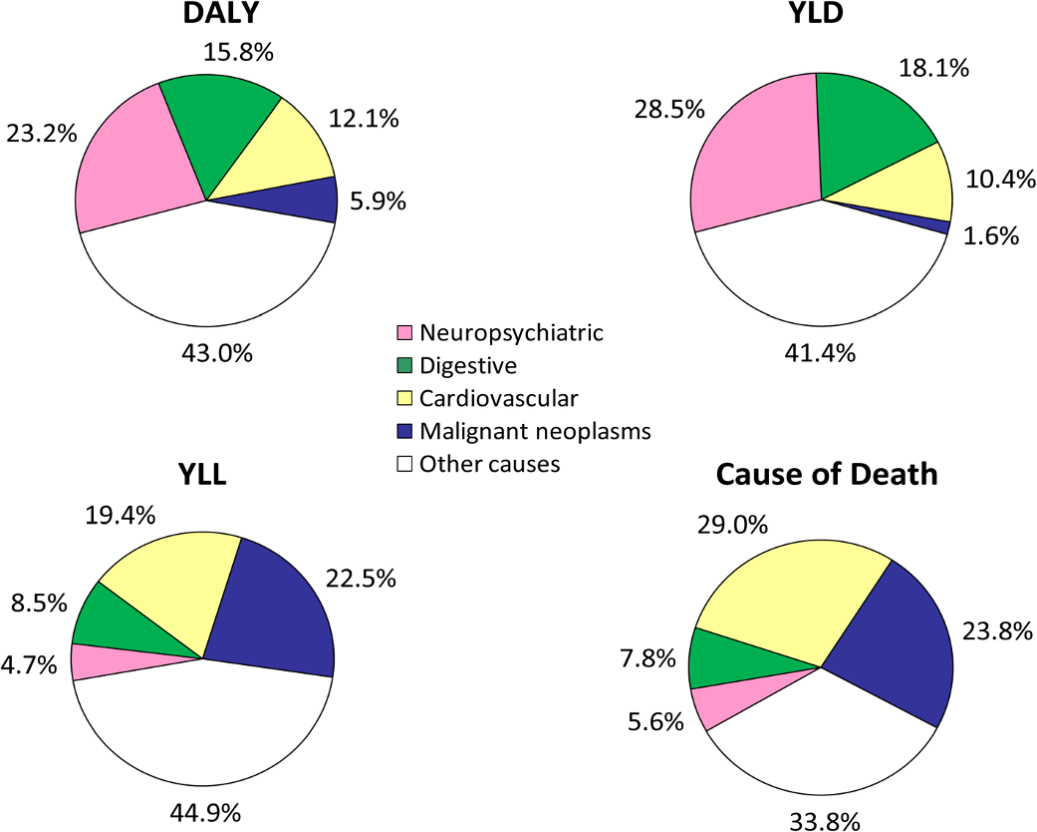
**Chronic disease burden in Chile.** DALY: Disability Adjusted Life Years; YLD: Years Lost due to Disability; YLL: Years Lost due to Death; DALY corresponds to the sum of YLD + YLL (Raw data source: Chilean Ministry of Health) [18].

In developed countries the economic impact of a disease is a major public policy issue. In Chile, where 23.8% of national mortality is due to cancer (Figure 2), we have calculated that Chile currently spends US2.100 million (1% GDP) on cancer care and treatment. Calculating the chronic disease burden in Chile we see that cancer accounts for notable proportions of Years Lost due to Disability (YLD) and Years of Life Lost due to premature death (YLL). Disability-Adjusted Life Years (DALYs) corresponds to the sum of YLD + YLL (Figure 2). Whilst no formal economic studies of the burden of cancer have been conducted in Chile, the authors of this paper have estimated that the national income lost to cancer per year DALYs is US$ 3.5 billion. This value was achieved by multiplying the calculated VLL (175,711) by an estimated per capita income of US$20,000. This suggests that cancer has a major economic impact in Chile. However, along with cancer, an aging population also brings an increase in neuropsychiatric problems that will only place further burden on the Chilean economy. The absence of integrated health economic studies is notable in Chile and in public policy terms this is a major deficit that needs to be urgently rectified.

### Stomach and gallbladder cancer: the Chilean phenomenon

Based on world statistics, the Chilean population presents notoriously higher than average mortality and incidence rates for both stomach and gallbladder cancers [27, 28]. Stomach cancer has historically been one of the principal causes of cancer related death worldwide. However, while rates have declined in westernized populations of North America, Northern and Western Europe, regions such as South America still post high mortality rates [29].

While stomach cancer is currently the most common malignancy in Chile, in the United Kingdom (UK), stomach cancer no longer figures as one of the ten most common cancers, with the incidence rate having fallen by over 60% since the mid-70s [30]. In 2009, Chile registered 3,350 deaths from stomach cancer giving a rate of 19.8 per 100,000 inhabitants [12].

As has been reported globally there is also a strong correlation between stomach cancer and the indigenous population [31]. This is also true in Chile, where between 1998 and 2002, a crude incidence rate of 29.2 per 100,000 inhabitants was reported in the city of Valdivia in the mid-southern region of the country [27]. Most stomach cancer patients are male, with urban residence and a low level of schooling [27, 32]. Along with social determinants and advanced clinical stage at consultation, infection with the bacteria *Helicobacter pylori* is also recognized as a principal risk factor for stomach cancer in the Chilean population [27, 32]. In addition, *Helicobacter* infection has been associated to lower socioeconomic structure. This observation is in line with statistics implicating the involvement of infection in 22.9% of cancers in lower income countries, as opposed to 16% globally [14, 33].

Another factor in the Chilean stomach cancer story is salt. In Chile, it is estimated that the average salt (NaCl) consumption is around 10.4 g per day in adults [34, 35].This value is significantly higher than in British adults who consume on average 8.1 g per day [36]. The interaction between salt, *Helicobacter* and genetic predisposition in the Chilean population are likely to underlie this high incidence. However, genetic factors aside, these above mentioned risk factors suggest that prevention, through health education and eradication of *Helicobacter pylori*, together with better screening procedures for precancerous lesions may well deliver a notable improvement in incidence and prognosis to the Chilean population.

While stomach pattern follows a worldwide trend in reducing as socioeconomic status increases, a statistic that is uniquely Chilean is the incidence of gallbladder cancer [37]. In the UK, gallbladder is considered a rare cancer, with 700 new cases and 438 deaths recorded in 2008 [38]. Chile recorded 1819 deaths from gallbladder and bile duct related cancer in the same year. Further emphasizing the gallbladder problem, in Chile the estimated incidence and mortality rates for this cancer are 13.4 and 11.5 per 100,000 female habitants, while in Latin-American these rate are 3.7 and 3.0, and in the USA 1.6 and 0.6 respectively [39]. The highest risk group for gallbladder cancer is among Amerindians, Mapuche and Hispanic women with less than four years of schooling [28]. On the other hand, the lowest standardized incidence rates were among Hispanic men and women with more than eight years of schooling. Thus, ethnic origin, low schooling, the female sex and urban residence were deemed independent risk factors. In the Mapuche population, the incidence of gallbladder cancer has reached 269.2 per 100,000 women in specific age groups [28]. Interestingly, cholelithiasis is always a precursor to gallbladder cancer, suggesting that screening programs and cholecistectomy could significantly decrease gallbladder cancer death. A pilot study of gallbladder cancer lead by the US-National Institute of Health (NIH) (Government trial identifier NCT01520259) in conjunction with Chilean Universities intends to recruit 120 cases to assess the feasibility of a full-scale population based multidisciplinary gallbladder cancer study [40]. Another recognized risk factor is the chronic carriage of *Salmonella typhi*, which would be the long-term effect of the hyperendemic of Typhoid Fever that occurred in Chile during the 1970s and 80s [41]. As Chile moves towards a more westernized lifestyle, the risk factors and incidence are likely to fall, however, the sheer numbers of gallbladder cancer cases in Chile suggests a genetic or environmental factor that is not being fully addressed by the nation’s scientific and medical community [42].

Another malignancy that demonstrates peculiar statistics in Chile is lung cancer. The majority of Chile presents slower than developed country incidence rates. However, the occurrence of high arsenic exposure in drinking water (>200 ug/L as opposed to now recommended <10 ug/L) from 1930 to 1977 in the Antofagasta Region in the north of the country and in particular levels of 860 ug/L in the City of Antofagasta between 1958 to 1970 has resulted in high lung cancer incidence and mortality [43]. Despite water filters and regulations being introduced in the 1970s, a significantly high odds ratio to develop lung and bladder cancer in this region still exists up until today [44]. However, not all cancers increased in mortality. A recent publication has speculated a rapid reduction in breast cancer mortality in the years during and directly following exposure to arsenic-contaminated drinking water in Antofagasta [45].

### Obesity and cancer in the Chilean population

In the National Health Survey of 2009, it was reported that over 67% of the surveyed population was overweight with 25% obese. Over 88% of the population was estimated to lead a sedentary lifestyle and an alarming statistic was the appearance of a significant number (3.3%) of morbidly obese females within the population [46, 47]. In neighboring areas within Chile’s capital Santiago, girls at private school underwent menarche six months later that girls at a state run school. Obesity in this case appears to be the smoking gun, with age of menarche correlating almost perfectly with body mass index [48]. Unsurprisingly, increased obesity numbers also correlated well with an increase in type-2 diabetes from 6.3% in 2003 to 9.4% in 2010 [48]. Interestingly and again demonstrating inequality, diabetes was three times more prevalent in members of the society with lower schooling [48].

Chile is now part of the Latin American Consortium of Studies in Obesity (LASO) [49] and it has also joined with the United Nations sponsored schemes on obesity control. The government statistics demonstrate that 24.5% of the men and 33.6% of women are obese, placing Chile together with Venezuela and Argentina in leading South America in this category. A recent publication suggests that 20% of Chilean cancers are directly related to obesity, with most affected being endometrial (47%) in women, and esophageal (35%) and pancreatic (31%) in men [39]. The authors of this publication conclude, again demonstrating Chile’s rapid incorporation of a westernized lifestyle, that the incidence of obesity-related cancers resembled the distribution of the USA and the UK more than developing countries [39]. Obesity-related health problems maybe confounded in coming years by the observation of increased gallstone incidence in obese and overweight children [50]. This, coupled to the observation that all gallbladder cancer patients first present gallstones, may further increase ChileÂ´s notorious burden of gallbladder cancer.

### Analysis to allow the development of a cancer healthcare system in an emerging economy

Despite persistent inequalities in cancer distribution, the Chilean population now has greater access to a public and private health system than a decade ago. Provision of services is mainly delivered by public sector services with an overlay of private providers under contract with public insurance system. The Ministry of Health is responsible for setting public policy in health including cancer prevention and mandatory strategies for infections, and chronic disease including cancer and mental conditions [51]. The 2005 Health Reform established a set of guarantees for access, care and quality for a significant number of health conditions including some cancers [52, 53]. This reform has impacted positively in coverage and equity in healthcare across the Chilean population, including cancer. On the delivery side, the public system runs a network of Primary Health care, with around 1500 points of service managed by municipalities in a decentralized mode. Regional Health care services administer 60 hospitals tasked with complex case management (tertiary), 100 less complex hospitals (equivalent to secondary or district level care) and a small number of highly specialized institutes (e.g. Neurosurgery, Cardio-respiratory, and Cancer) [54]. Currently, Chile only offers first line therapy within the public system, and there is also a need to re-consider the overall quality and delivery of radiotherapy, surgery and pathology to assess what Chile’s essential cancer guidelines should or should not include.

Chile currently possesses 60 oncologists (*data kindly supplied by the Chilean Society of Medical Oncology*), a number clearly inadequate to meet the pending cancer burden. To achieve the same coverage of oncologists per capita currently present in the USA, Chile would have to increase their number of oncologists to over 500. This statistic mirrors the overall observation that Chile possesses only 1.6 medical doctors per 1000 inhabitants as opposed to the OECD average of 3.2 medics per 1000 inhabitants.

### Clinical trials

In the clinical trial registry at the Chilean National Institute of Health (ISP Chile), 65 cancer clinical trials were active or in progress in 2014 [55]. Most of them (76.9%) are phase II/III or III and only 4.6% are in early phases (IB or I/II). The majority support comes from outside the country with Roche accounting for 10% of trials, Pfizer 7.6%, Bristol-Myers Squibb 7.6%, Merck Sharp and Dohme 6.6% and Amgen 6.6%. Through GOCCHI (Grupo Oncológico Cooperativo Chileno de Investigación) [56], a Breast International Group member, Chile is involved in multicenter trials such as the US-LA-CRN (United States-Latin-American- Cancer research Network) trial looking into the molecular profiling of stage I and II breast cancers in Latin-America and the worldwide ATLAS trial (Adjuvant Tamoxifen: Longer Against Shorter) [57, 58].

### Cancer registries, tumor banks and legislation

Chile is in the initial stages of establishing and implementing a regulatory legal framework in biomedical scientific research. Chile is in transition from technical standards, focused exclusively on clinical trials (Technical Standard No. 57 of 2001), to specific legislation encompassing all biomedical research (Law No. 20120 of 2006 [59] and its Regulatory Decree No. 114 of 2010 [60], as amended in 2013 [61]). However, there are imperfections in this legal framework that require urgent clarification to permit biobanking, biomedical research and the participation of Chilean subjects in clinical trials.

Chile currently possesses a national registry of pediatric cancers [62] and three established regional cancer registries. The established registries, two of which are IACR (International Association of Cancer Registries) registered [63], provide incidence data from populations in the North (*Registro de cancer de Antofagasta*) [64] and South (*Registro de cancer de Valdivia and Bio-Bio*) of the country [65, 66]. Together with the established registries, there are three registries in development (*Región de Arica-Parinacota, Región del Maule, Provincia de Concepción*), which when fully operational should give vigilance of 18.5% of the country. Despite persistent underfunding, often only having one part-time worker, these six databases have provided key information about the most frequent cancers in the country. However, approximately 40% of the Chilean population is centralized in the capital Santiago, and there are no immediate plans to monitor cancer incidence in this region.

Through involvement in clinical trials such as the above mentioned US-LA-CRN, Chile created its first centralized biobank for breast tumor samples. The concept and requirement for tumor banking in Chile is gathering momentum, however, while public universities and several private universities and clinics have started to implement banking systems, there is no collective consensus on methodology and little interaction between the players. This is principally due to a lack government priority, regulatory confusion on the legal aspects of sample taking and a scarcity of funding.

## Review and analysis of public policy approaches to cancer in Chile

There is no National Cancer Policy in Chile. Nevertheless, the Ministry of Health has established four programs relating to cancer in the last 25 years. The four programs are cervical cancer, established in 1987, anti cancer medicines for children and adults in 1988, breast cancer in 1995 and palliative care in 1995. The Health Reform of 2005 also established Explicit Guarantees for Free Access (GES) [67] in 15 conditions related to cancer [52, 53]. Secondary prevention is included for four of these malignancies: cervical cancer –Pap smear every three years from ages 25 to 64, breast cancer–one mammography at age 50 years, stomach-endoscopy for symptomatic patients over age 40, and cholecystectomy to prevent gallbladder cancer in people aged 35 to 49. Furthermore, the public health system offers diagnoses and treatment for testicular, prostatic, lymphomas, palliative care and all cancer in children under 15 years of age. In 2014 Chile passed an amendment to the Labor Code, (new Article 66a) that states that female workers over 40 years and male workers over 50 are entitled to half a working day to undergo preventive medicine exams (mammography, prostate exams, and Papanicolau were specifically mentioned) [68, 69]. Despite these programs, screening coverage is still below the gold standard.

Chile’s neighbors Argentina and Peru [70], as in higher income countries [71], offer several lines of chemotherapy to cancer patients, while Chile is only now coming close to full national coverage for first line therapies and implementing the possibility of second line therapy. Several prominent cancers in the country, such as lung cancer and stomach cancer still do not have chemotherapy treatment covered in the nation health plan. The Chilean Minister of Health is currently preparing a legal initiative to cover high costs drugs for low frequency diseases [72]. These pathologies correspond to autoimmune disease and metabolic disorders among others, however, this initiative may well path the way for future high cost cancer therapies.

### Analysis of cancer research in Chile

With excellence in care and improved outcomes so dependent on having a learning cancer care system, the state of R&D is a critical determinant of current and future quality in any national cancer control system.

**Figure 3:**
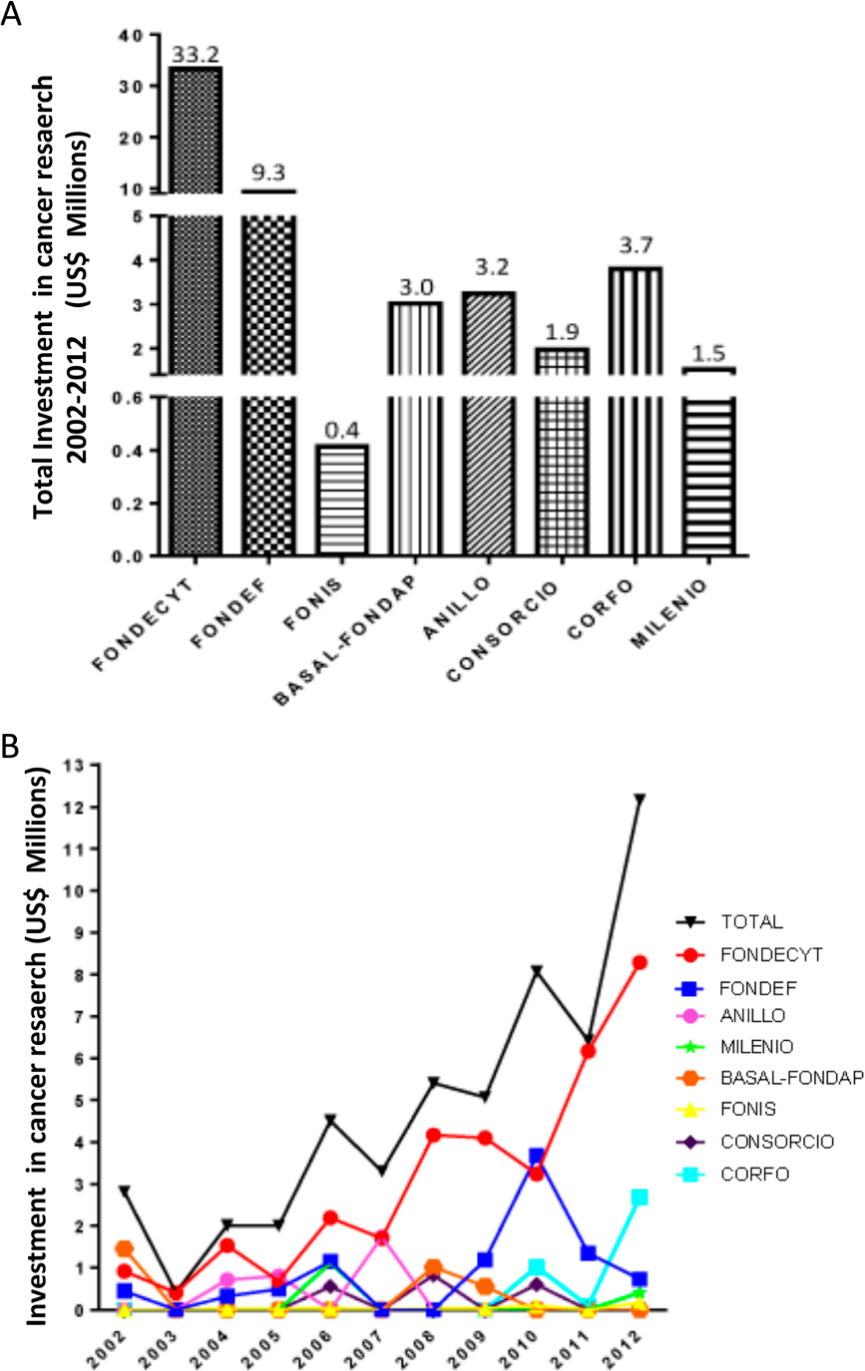
**State-subsidized investment in cancer research in Chile. A)** Total Investment in cancer research 2002–2012 (US$ Millions). **B)** Investment in cancer research (US$ Millions) by year between 2002 and 2012 according to public funding source.

Scientific research in Chile has been historically supported by CONICYT, which is under the jurisdiction of the Ministry of Education. In the time period of 2002–2012 Chilean state funds invested approximately US$55 million in cancer research (Figure 3A). The majority of these funds (US$33.2 million) came from the basic science grants awarded by FONDECYT, with the more applied Ministry of Education FONDEF grants contributing US$9.3 million. FONDECYT grants have been rising steadily with the number of new researchers taking up academic positions (Figure 3B). In 2002 seven Regular-FONDECYT grants (USA RO1 equivalent) were awarded with an average of US$ 42,000 per year for 3 years. In 2012 seventeen Regular-FONDECYT grants with an average of US$ 100,000 per year were awarded. In the last decade FONDECYT has also initiated an early investigator grant (FONDECYT Inicio) that in 2012 awarded 5 cancer research projects with an average of US$ 41,000 per year over 3 years. Encouragingly, since 2011 the funds coming from the Ministry of Economy (economic development board, CORFO) have started to rise substantially. This is principally due to the increased emphasis that CORFO has recently placed on innovation through science. Although not all the figures are currently available to incorporate into the graphs, this CORFO investment has continued to rise in 2013 and 2014. In summary, the overall statistics demonstrate a steady increase in cancer funding which is generally in line with the number of new laboratory heads choosing to dedicate their careers to cancer research. However, the figures are still low when compared to other nations. Chile spent US$12 million in cancer research in 2012, while the National Cancer Institute (NCI-USA) budget was US$ 4.9 billion for the same period.

**Figure 4:**
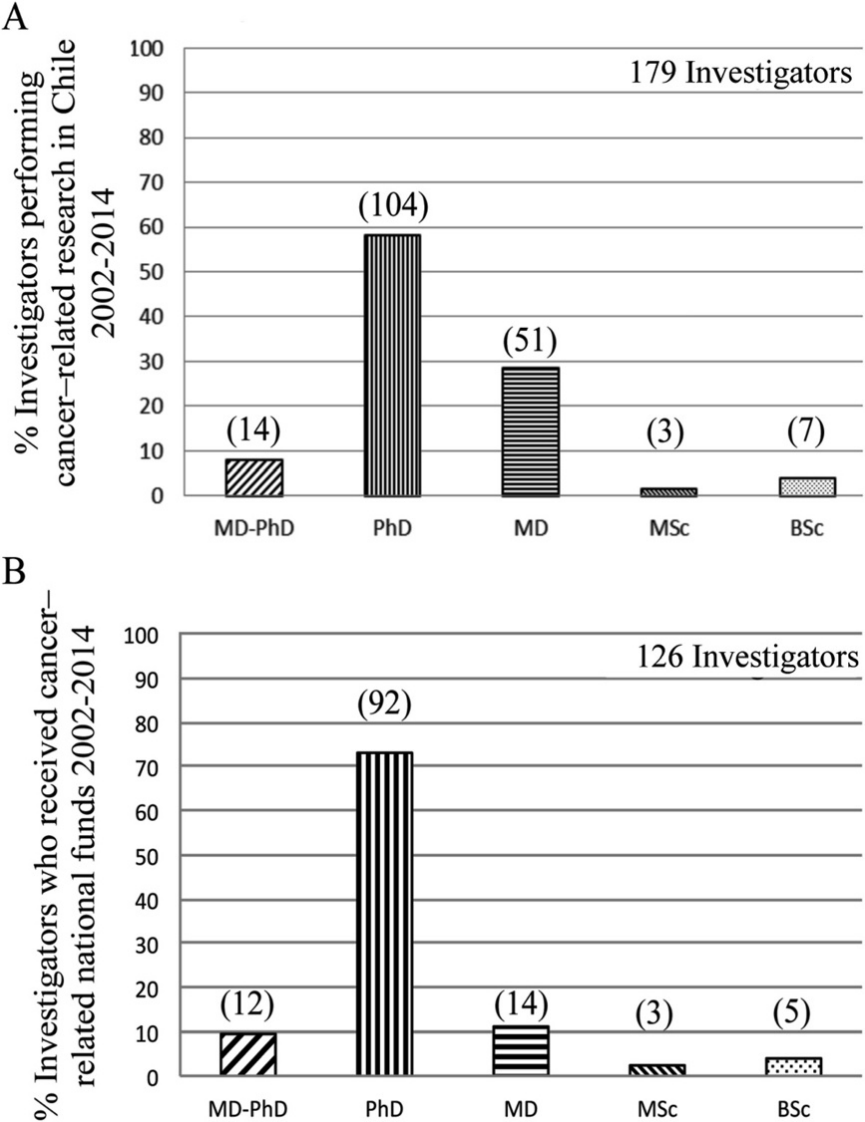
**Quantification of cancer researchers in Chile. A)** Total number of investigators, separated into highest academic obtained, that have performed cancer research in Chile. **B)** Total number of investigators, separated into highest academic obtained, who have received national funds for the purposes of cancer research in Chile.

Chile currently possesses 179 cancer investigators (Figure 4A). A closer examination of human resources demonstrates 126 investigators have been directly awarded grant funding. Breaking down the investigators with funding into highest degree obtained, we see that that 12 are MD/PhD (9.5%), 92 are PhD (73%), 14 are MD (11%), 3 MSc (2.4%) and 5 BSc (4%) (Figure 4B). Of the 65 medical physicians questioned, 29 did not receive public funding but participated work in international pharmaceutical company projects (principally phase III trials) or intradepartmental clinical protocols.

**Figure 5:**
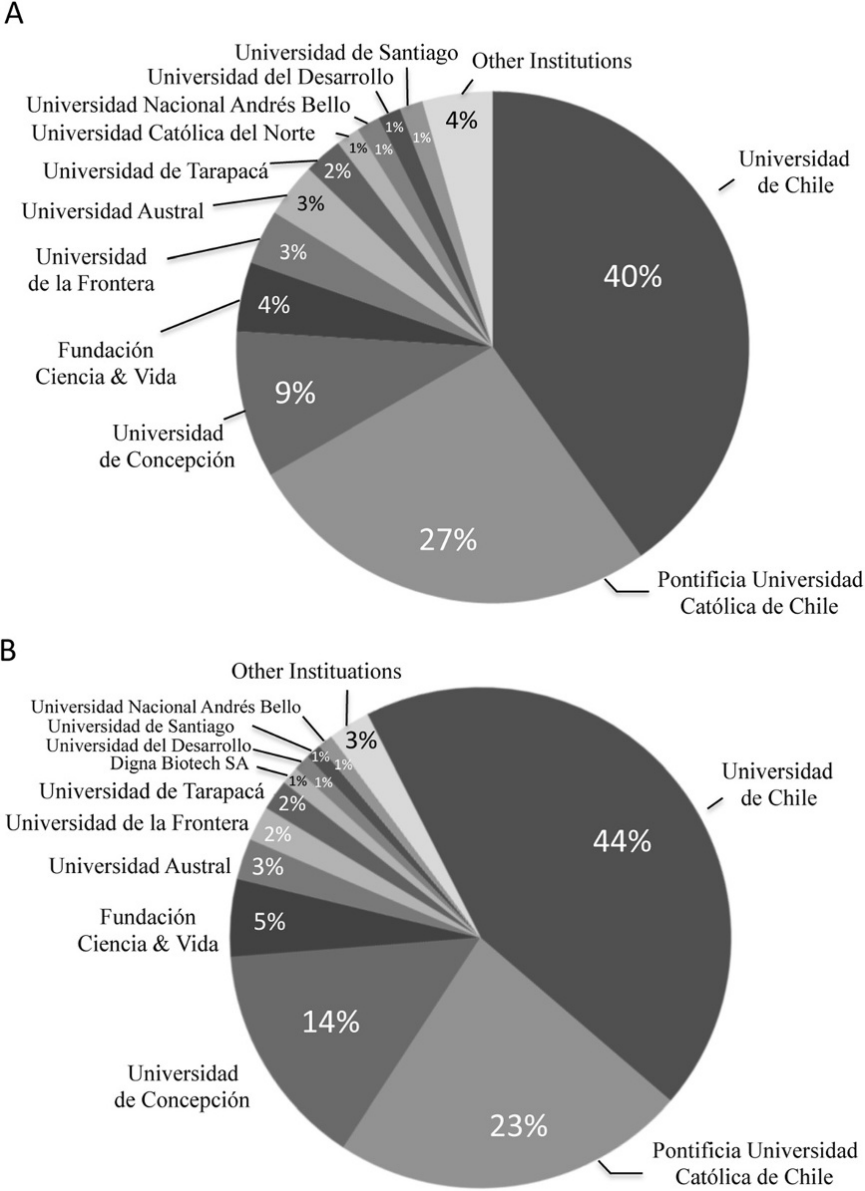
**Location of cancer research in Chile. A)** Institutional affiliation of cancer-related investigators in Chile. **B)** Direct national funding received by cancer-related investigators according to institutional affiliation (expressed as percentage of total funding obtained between 2002–2012).

The distribution of cancer investigators is centered on two universities; the University of Chile (*Universidad de Chile*) and the Catholic University of Chile (*Pontificia Universidad Católica de Chile*), both located in the capital Santiago (Figure 5). These two universities between them house 52% of national investigators and receive 67% of national oncology funding (Figure 5A and B, respectively). Both universities possess their own hospitals and a network affiliated medical institutions offering cancer treatment and care. In the last world university ranking survey (http://www.topuniversities.com/), the Catholic University of Chile was ranked 1st in Latin America and 166 worldwide, while University of Chile was positioned 6th in Latin America and 223 worldwide. Chile’s largest regional university, the southern University of Conception (*Universidad de Concepción*) houses 9% of the country’s cancer investigators and receives 13% of national oncology funding. Despite the classical dominance of these three universities in all areas of scientific discipline in Chile, in recent years other principally private universities have started to establish cancer centers and attract highly qualified investigators. Notably, the *Universidad Nacional Andrés Bello, Universidad del Desarrollo, Universidad Mayor, Universidad de Los Andes, Clínica Las Condes* and *Fundación Ciencia & Vida* (Science & Life Foundation) have investigated heavily in the field of oncology and may well compete with the leading national universities in the not so distant future.

**Figure 6:**
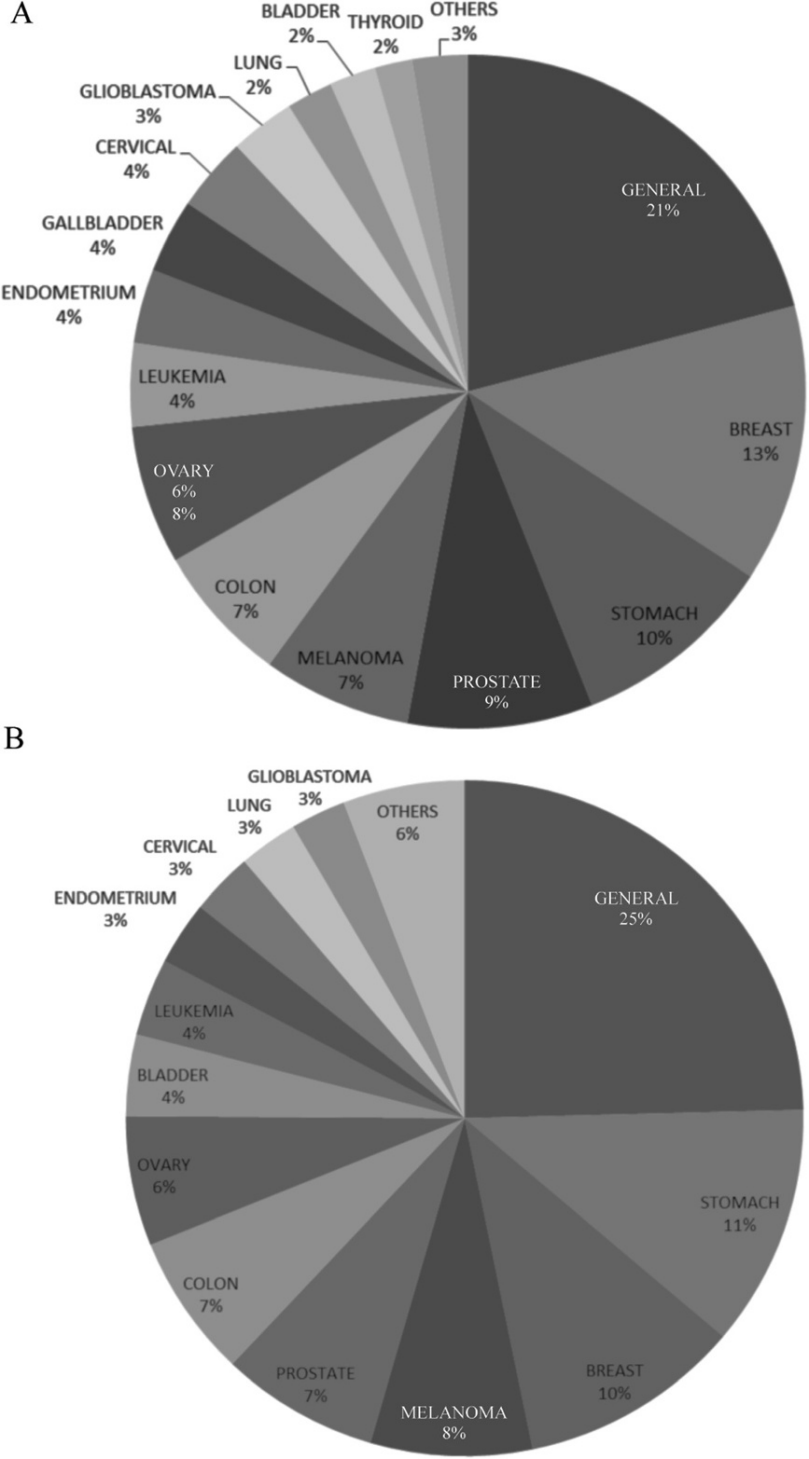
**Areas of oncology research in Chile. A)** Research categories of cancer-related projects for investigators who have received national funding or participated in national or international clinical trials and protocols. **B)** Research categories of cancer-related investigation based on financial allocation from national funds between 2002 and 2012.

Breaking down the specific areas of oncology research we see a similar pattern to that observed in many other countries, where 27% of the funding is not designated to a particular cancer type but utilizes cancer cell models (represented as *General* in Figure 6A and B). Breast, colorectal and prostate cancer between them account for 29% of the researchers and funding. Of cancers with predominant incidence in Chile, only stomach cancer receives sizable funds (10%), while lung cancer receives only 2% and gallbladder cancer 4%. In other words, the cancers responsible for the majority of lives lost to cancer receive only 16% of national funding. This finance figure roughly reflects the number of researchers dedicated to these cancers and thus the problem maybe manpower and not funding priorities. A possible explanation for the shortage of gallbladder cancer research is the training that the researchers received as post-doctoral fellows outside of Chile. The cancer-types studied upon returning (or arriving) to Chile tend to be the malignancies studied in North America or Europe, thus a high emphasis on breast and prostate cancer and a low percentage of stomach and gallbladder cancers. The authors suggest an intervention from the Ministry of Health and/or funding agencies to put an emphasis on gallbladder, stomach and lung cancer. Epidemiological, access to medical care and preventative research needs to be conducted hand in hand with the cellular biology, genetic and epigenetic aspects of this cancer, especially in relation to environmental factors.

An unexplained phenomenon in Chilean cancer research is the lack of attention to lung cancer. Lung cancer is one of the most funded cancers by the NCI-USA and one of the biggest killers in Chilean society, yet accounts for only 4% of national researchers and 3% of funding. This statistic may now change as the pharmaceutical company Pfizer has recently been adjudicated funds by CORFO to investigate bioinformatic components of South American lung cancer patients [73].

### Analysis of scientific productivity in Chilean cancer research

**Figure 7:**
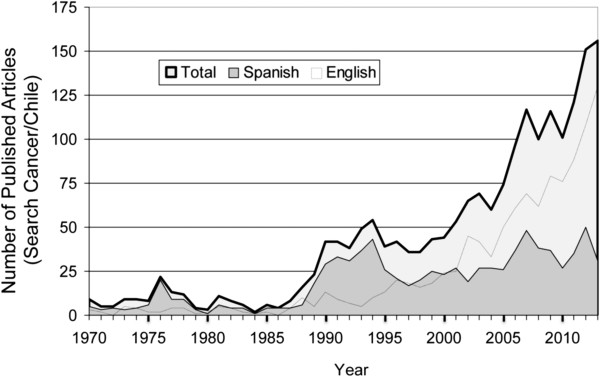
**Chilean cancer publication record (1970–2013).** Trends in Chilean scientific cancer-related publications (written in English and Spanish), as indexed in PubMed database.

Productivity can be measured in the publications, patents and the formation of human resources. Analysis of the current cancer publication record in Chile shows an upward trend since the late 1980s with switch from Spanish language to English language ISI-indexed journals since the year 2000 (Figure 7). Encouraging, the increase in cancer funding (Figure 3B) mirrors well the increase in cancer-related publications, demonstrating that increased financial investment is delivering higher scientific productivity. Increased scientific funding has also permitted an increase in doctoral students trained at Chilean Universities since early 2000. Chile now boasts a steady flow of doctoral students, albeit low in comparison to high-income countries. For example, the Catholic University of Chile has graduated more doctoral students in the last decade that in the 127 year history of the university (personal communication with university authorities). Other public and private universities are following suit, while the government has implemented schemes to finance doctoral theses in centers of excellence outside the country [74]. As a result, at Chile’s highest attended science meeting, *The Annual Meeting of the Chilean Society for Cellular Biology* (approximately 700 participants), cancer-related presentations have risen from 1% to 16% during the last decade.

While many graduated students chose to stay in Chile, a high percentage has undertaken at least one doctoral fellowship in North America or Europe before returning to academic positions in Chile. However, this encouraging momentum may now start to slow, as long-term investment in science is not creating new academic positions for returning PhDs. Thus, as seen in other emerging economies, there is a brain drain where students are trained at the cost of Chilean taxpayer to benefit principally the research and economy in North America [75]. Confounding this problem is the hesitancy of the Chilean private sector to incorporate PhDs and thus academia is often the only viable career path for doctoral graduates. The culture in Chile for the private sector (mainly pharmaceutical sector) to take an active role in research did not exist a decade ago. The previous fragility of the Chilean marketplace made the usual minimum 10-year return on investigation, a horizon too distant to be considered economically tangible. However, by requesting private-sector partial funding and placing emphasis on research that may result in patenting, CONICYT and CORFO have made available additional public funds, some of which have gone into cancer initiates. Halfway through the last decade the leading Chilean pharmaceutical company, *Corporación Farmacéutica Recalcine* (purchased in 2014 by Abbott Laboratories), started to co-sponsor cancer-related investigation at the leading universities. Although still few in number, other companies have since followed their lead in the research field. Andes Biotechnology, a Chilean company formed from as a spin-off of Chile’s first Science and Technology Park (*Fundacion Ciencia & Vida*) and the private *Andrés Bello University*, is now able to boast the first significant commercialization stemming from Chilean based cancer research [76]. In the future, tax breaks and incentives could stimulate the private sector as a whole to invest in research both for innovation of new products and in the improvement of the processes within their own manufacturing pipeline. A new law providing tax concessions to private sector companies investing in R&D was introduced in September 2012 [77].

A further parameter to measure investigation in cancer research is the generation of intellectual property. Until mid-2014 there have been 54 individual patent fillings related to cancer from Chile, with over half of these patent applications corresponding to new products. Since the culture of patent applications has arrived late to the Chile, this figure reflects well upon the translational-orientation of national cancer investigators.

## Conclusions

### Cancer in transition: next steps for an emerging economy in addressing cancer control

Chile, a country split into two unequal worlds of public and private medicine, must work to guarantee fairness in its cancer outcomes, as well as promote a national cancer research system. Chile has a rapidly aging population wherein 40% smoke, 67% are overweight, 18% abuse alcohol, 88% observe a sedentary lifestyle, and thus the corresponding future burden of cancer will have a serious negative impact on the Chilean society and the ability to deliver an affordable health care system [11]. Accumulating epidemiological evidence confirms that the socioeconomic divide in Chile is a major factor in cancer incidence and mortality and that this inequality is most notably manifested in stomach and gallbladder cancers. Prime suspects for the culpability of this socioeconomic divide are access to the health system, lack of early detection, sanitary conditions, working conditions and habits such as alcohol, tobacco and diet. This striking social division for stomach and gallbladder cancers highlights the need for education and investigation to reduce the growing inequality. We suggest that a future strategy for Chilean universities may well be to initiate academic positions for investigators specializing in stomach, gallbladder and lung cancer. Furthermore, we suggest the initiation of scientific task force to understand at the epidemiological, genetic and cellular biology level the disproportionate incidence of gallbladder in Chile. The task of the Ministry of Health must be to define how and within a hypothecated budget, Chile can address this major public health issue in terms of sustainable infrastructure, prevention strategies, and cost effective care pathways. To address this issue, the authors have outlined in Table 1 (*Key cancer public policy framing for the Chile economy*) their recommendations for Chile to tackle the cancer burden. Although geared specifically to Chile, this table may well offer a guideline for many other emerging economies faced with a complex burden of cancer. By far the greatest return on investment will be public education and prevention, followed by screening and early detection. The future action of Chile in the face of the general international failure of population wide mammograms [78] and prostate specific antigen testing is a challenge [79, 80].

**Table 1:** **Key cancer public policy framing for the Chilean economy**

•	Publicly recognize the imminent burden of cancer and implement a strong and visible National Cancer Policy. Stimulate pro-equity interventions in poor areas of the country.
•	Introduce a national cancer law. Place cancer policy on the political and public agenda and permit top-down implementations of cancer policy in the country.
•	Create integrated cancer hospitals. To start, these establishments should be in the North (possibly Antofagasta), South (possibly Concepcion or Valdivia) and in the capital city of Santiago. The National Cancer Institute (*Instituto Nacional de Cáncer*) in Santiago would be the prime candidate to transform into an integrated cancer hospital.
•	Conduct regular systemic nationwide studies on the status of oncology at all levels. Perform regular evaluations of the impact of health and cancer reforms. Promote studies of the economics burden of cancer to inform a National Cancer Policy.
•	Initiate nationwide prevention programs incorporating all sectors of society. This should include stronger than the current emphasis on tobacco use, salt consumption, increase consumption of fruit and vegetables and decrease obesity, especially at schooling age. Promote research into prevention strategies and introduce public demystification and education in cancer, starting at the schooling age.
•	Promote the investigation in cancers of high national relevance (e.g. stomach, gallbladder and arsenic-related lung cancer in Chile). We suggest the setting up of specific task forces promoting multi-institutional team science. Lifting any import tax on reagents and literature destined for education and scientific research.
•	Partner with other South American countries to pool resources on regional problems. Reach out to North American and European countries for specialist help and *off the shelf* solutions to cancer-related problems.
•	Specifically focus on clinical trial promotion to stimulate pharmaceutical company interest in Chile and clinical oncologist participation in research.
•	Promote incentives and tax breaks to further stimulate the private sector investment, coupled with stimulation of capital risk and angel investments in the biomedical field. Clarify national finance laws to allow institutions to receive legacies and personal/company donations for cancer-related activities.
•	Create, strengthen and expand of regional cancer registries. Evaluate the implementation of cancer registries to incorporate the capital city, Santiago. Promotion of tumor banking and other national infrastructure initiatives. The encouragement of existing initiatives to unite under a virtual national network. Redefine the existing legal framework for the use of human samples and clinical information, as to not impinge on biobanking and medical research.
•	Promote the formation of medical cancer specialists (e.g. oncologists, palliative care, nursing and other cancer-related human resources).

Confounding this dilemma is the change in cancer treatment from standard care to personalized medicine. There will be a burden on the Ministry of Health to adopt these new and expensive therapies. The last few years have seen Chile implement the breast cancer drug Trastazumab (Herceptin™) under the government financed health plan. It could be reasonably argued that the implantation of this treatment came about due to public demand, in particular from breast cancer sufferers and their families. With Chile’s continued growth and future prosperity it will be morally and politically unacceptable not to provide a national cost-effective cancer care system. Thus, infrastructure affordability, not just in cancer treatment medicines, but also in the ever-updating cancer technologies such as imaging, will need to be addressed. On the other side of the coin is the danger of taking on too many low-gain, high-price technologies. There is an important need to make public policy in this area evidenced based, affordable and fair to prevent unconstrained exploitation by private interests. Equity must be a central part of developing cancer public policy and planning.

Chile now has the infrastructure, the economy and the human capacity within the scientific community to perform world-class cancer research. PhDs or MD now conduct national cancer research and thus conditions are clearly strong for additional investment in investigation. Overall positive trends in cancer outputs is a strong metric that investing more in cancer research in terms of public policy will generate returns on investment through knowledge and innovation. Moreover, the mostly ISI-indexed English language publications now heavy outweigh publications in Spanish language journals (another metric of international quality). However, despite these positive trends the current investment (public and private) is clearly insufficient to address the magnitude of the problem, as only a small percentage of the already scarce resources are invested in cancer research. Currently, the problem in Chile is not principally the number of grants available, as the basic research grant (FONDECYT Regular) is now awarded to one in two applicants. The problem is the size and delegation of the grants. Chilean researchers also face the additional problem of not having any local production of scientific agents and suffer substantial taxation on any imported product destined for scientific research.

While cancer policy should be initiated and delivered by federal bodies, it is the grass roots of the medical cancer and academic organizations that should drive public policy. As many emerging economies pass through the period of transition that Chile currently faces, it is important for them not to try and reinvent the wheel, but instead look outside for advice and guidance. There currently exist several international collaborations driven by the Chilean cancer community, notably with the NCI/NIH, IARC, MD Anderson, the University of California and the King’s Health Partners, London. These and other strategic collaborations can play an important role in bringing Chile quickly up to speed, through the implementation of *off the shelf* solutions to promote cancer care and research infrastructure.

To achieve necessary changes, the impetus must come initially from the Ministry of Health, with a public declaration of intent to create a National Cancer Plan. Currently, there is an evident asymmetry between the magnitude of the cancer problem and important variables such as policy, public awareness and equity. Although private-public discussion initiatives such as the National Cancer Forum have been recently created [81], more effort in human resources, planning, policy aspects and institutional development is urgently required; and this will need to be cross departmental, an additional challenge. It is important to highlight the magnitude of cancer to Chilean society through all possible means, preceded by a national social interest study. The analysis presented in this work clearly shows that Chile is ready and needing of a National Cancer Policy. Such a plan also needs a robust national cancer intelligence system. However, plans for such registries, already drawn up by the Ministry of Health, unfortunately are not viable to define a strategic priority. This clearly needs to change. The analysis of cancer statistics, a clear vision of the trends and distribution of cancer and the socio-economic needs in Chile, are fundamental if substantially increased public funds are going to be spent in care. Hand in hand with care, is the need to put more funding into cancer research as well as to take an interdisciplinary approach to integrating basic scientists and medical community, healthcare professionals, social scientists and economists. For Chile, as well as all other emerging economies, it is clear that a radical shift in cancer public policy is required.
